# The Association Meetings for 1883

**Published:** 1883-07

**Authors:** 


THE ASSOCIATION MEETINGS FOR 1883.–The annual meetings of the principal dental associations of the country will be held during the latter part of the present and the beginning of the month of August. Themeeting of the Southern Dental Association will commence July 31st 1883, at Atlanta Georgia, and that of the American Dental Association at Niagara Falls, August 7th, 1883. Both of these meetings promise to be of great interest and benefit to all who may attend them, and be to largely attended by the representative members of the profession. The annual meeting of the Georgia State Dental Society will be held at the same time as the Southern meeting, and these joint meetings no doubt will add considerably to the number who will be present at Atlanta. It is proposed to hold meetings of State examining boards, representatives of the different dental educational institutions, etc., etc., during the meeting of the American Dental Association, which will no doubt add to the interest of the occasion.

The value these annual meetings cannot well be overestimated to the dental practitioner, especially to those who are more or less isolated from their brethren owing to country location. The great mistake, however, often made at such meetings is the valuably time consumed in the consideration of abstruse theories, which had better be discussed through the pages of the Journals, or in a Society formed for the purpose of scientific investigation, the results of which could be reported at the annual meetings and thereby allow more time to be devoted to such practical matters as would directly interest every practicing dentist in the country. Were such a course pursued at our annual meetings the effect would be to draw many more practitioners from obscure localities who would be assured of instruction on just such points as they could put into daily practice at home. This suggestion is worthy of attention, as the object of these annual meetings is to do the greatest possible amount of good to the profession generally.

